# Predator-induced defences in *Daphnia longicephala*: location of kairomone receptors and timeline of sensitive phases to trait formation

**DOI:** 10.1242/jeb.124552

**Published:** 2015-09

**Authors:** Linda C. Weiss, Julian Leimann, Ralph Tollrian

**Affiliations:** 1 Environmental Genomics Group School of Biosciences, University of Birmingham, Birmingham B15 2TT, UK; 2 Department of Animal Ecology, Evolution & Biodiversity, Ruhr-University Bochum, Universitätsstraße 150, Bochum 44780, Germany

**Keywords:** Chemoreception, Crustacea, Aesthetascs, Infochemical, Notonecta

## Abstract

The freshwater crustacean *Daphnia* adapts to changing predation risks by forming inducible defences. These are only formed when they are advantageous, saving associated costs when the defence is superfluous. However, in order to be effective, the time lag between the onset of predation and the defence formation has to be short. *Daphnia longicephala* develop huge protective crests upon exposure to chemical cues (kairomones) from its predator the heteropteran backswimmer *Notonecta glauca.* To analyse time lags, we determined kairomone-sensitive stages and the developmental time frames of inducible defences. Moreover, we looked at additive effects that could result from the summation of prolonged kairomone exposure. Kairomones are perceived by chemoreceptors and integrated by the nervous system, which alters the developmental program leading to defence formation. The underlying neuronal and developmental pathways are not thoroughly described and surprisingly, the location of the kairomone receptors is undetermined. We show that *D. longicephala* start to sense predator cues at the onset of the second juvenile instar, defences develop with a time lag of one instar and prolonged kairomone exposure does not impact the magnitude of the defence. By establishing a method to reversibly impair chemosensors, we show the first antennae as the location of kairomone-detecting chemoreceptors. This study provides fundamental information on kairomone perception, kairomone-sensitive stages, developmental time frames and lag times of inducible defences in *D. longicephala* that will greatly contribute to the further understanding of the neuronal and developmental mechanisms of predator-induced defences in *Daphnia*.

## Abstract (precis)

**Highlighted Article:** Chemoreceptors for kairomone perception are localized on the first antennae of *Daphnia longicephala* and their role in inducible defences contributes greatly to our understanding of the neuronal and developmental mechanisms of predator-induced defences.

## INTRODUCTION

Phenotypic plasticity describes the ability of an organism with a given genotype to develop alternative phenotypes in response to changing environmental conditions (Bradshaw, 1965; Brown et al., 1970). By adjusting their phenotype, organisms maximize their fitness in response to changing environmental factors (DeWitt and Scheiner, 2004), e.g. predation. In turn, prey organisms express defences that oppose the current predatory threat and increase survival chances of the individual. These, so-called inducible defences can manifest as behavioural, morphological or shifts in life-history parameters and are elicited upon the perception of predator-specific chemical cues called ‘kairomones’. All of these inducible defensive strategies incur costs that are saved when the defence is not essential (Auld et al., 2010; Barry, 2002; DeWitt, 1998; Tollrian and Dodson, 1999; Walls et al., 1991). In addition, costs are kept at a minimum, as many species scale their defences to the predation risk and the magnitude of defence formation is directly correlated with the concentration of predator kairomones (Tollrian, 1993). Kairomones, which are advantageous for the receiver but not the sender, are produced and released by the feeding predator (Laforsch et al., 2006). Most importantly, in order for inducible defence strategies to be advantageous, the costly time lags between kairomone perception and defence formation need to be minimal (Clark and Harvell, 1992; Jeschke et al., 2008; Hoverman and Relyea, 2007; Gabriel et al., 2005).

The ecological and evolutionary aspects of inducible defences have been thoroughly studied in the model freshwater crustacean *Daphnia* (Tollrian and Harvell, 1999)*.* Prominent examples of morphological defences include neckteeth development in *Daphnia pulex* induced by the phantom midge larvae *Chaoborus* (Diptera) (Krueger and Dodson, 1981; Tollrian, 1993) and backswimmer (*Notonecta*, Heteroptera)-induced crest development in *D. longicephala* (Grant and Bayly, 1981; Barry, 2000). These neckteeth have been shown to impose handling difficulties on the predator and hinder successful prey consumption, which increases prey survival chances (Jeschke et al., 2002).

The development of defences requires a series of successive biological reactions in which the nervous system perceives, integrates and transforms the kairomone signal into developmental changes and respectively initiates endocrine signals when life history is shifted (Barry, 2002; Miyakawa et al., 2010; Weiss et al., 2012). However, the exact pathways underlying kairomone perception remain to be determined. Astonishingly, the location of chemical cue perception, which is fundamental for the understanding of the neuronal mechanisms of inducible defences in *Daphnia*, is unknown. In cladocera, chemoreception is ascribed to the sensory papillae of the first antennae (Ekerholm and Hallberg, 2002; Hallberg et al., 1992). However, because of the small size and the extreme inaccessibility of the *Daphnia* first antennae, neither physiological nor electrophysiological proof has been provided to date.

Kairomone-sensitive periods are phases in which an organism is receptive to a specific type of environmental stimuli. These, and the developmental time frames that follow, have only been reported for neckteeth expression in *D. pulex* (Imai et al., 2009), where kairomone sensitivity is confined to embryonic and post-embryonic periods and neckteeth expression to juvenile developmental time frames.

In contrast to *D. pulex*, where the 1st juvenile instars have to be protected against a tactile predator selecting small prey, *D. longicephala* need to protect later instars against its visually hunting predator, which selects relatively large prey. Consequently, tiny neckteeth need to develop rapidly in *D. pulex*, whereas morphological features of *D. longicephala* are relatively large and possibly require a longer period to develop. Here, we performed experiments in *D. longicephala* to (1) define kairomone-sensitive periods, (2) determine the developmental time frames, (3) quantify the time lags between kairomone detection and defence-structure expression, as well as (4) measure potential additive effects from prolonged kairomone exposure. Using a defined kairomone induction assay, we found that crest development in *D. longicephala* does not start before the 4th juvenile instar. Additionally, the onset of defence development requires one instar i.e. two moulting cycles following kairomone exposure. In summary, *D. longicephala* sensitivity to the kairomone begins in the 2nd juvenile instar. Morphological defence development is limited to time frames covering later instars, with the earliest onset in the 4th juvenile instar. Defences are developed with a time lag of one instar.

Impairment of the first antennae in the presence of kairomones, leads to the inhibition of crest development. Our study provides fundamental insight into the neuronal and developmental characteristics of crest development in *D. longicephala*. This opens up new avenues for the investigation of the molecular mechanisms of predator detection, signal integration and transformation into a developmental response that ultimately results in environmentally adapted phenotypes.

## RESULTS

During our full factorial experiment – exposing *D. longicephala* to *Notonecta* kairomones in successive instars (1st, 2nd, 3rd, 4th, 5th) – development of defensive morphological traits (i.e. body length, spine length, crest width and crest height) was monitored in the 2nd, 3rd, 4th, 5th, 6th and 7th instar. Animals moulted approximately every 24 h until the 4th juvenile instar. Subsequent instars have a longer duration of approximately 48 h. Animals deposited their first clutch after another 48 h and their second clutch 2 days later. *Notonecta*-exposed and control animals reached maturity on the same day.

### Timeline of sensitive phases and trait formation

Using our induction scheme, we aimed to determine the onset of kairomone sensitivity, developmental time frames, time lags and additive effects of defence development.

**Fig. 1.**
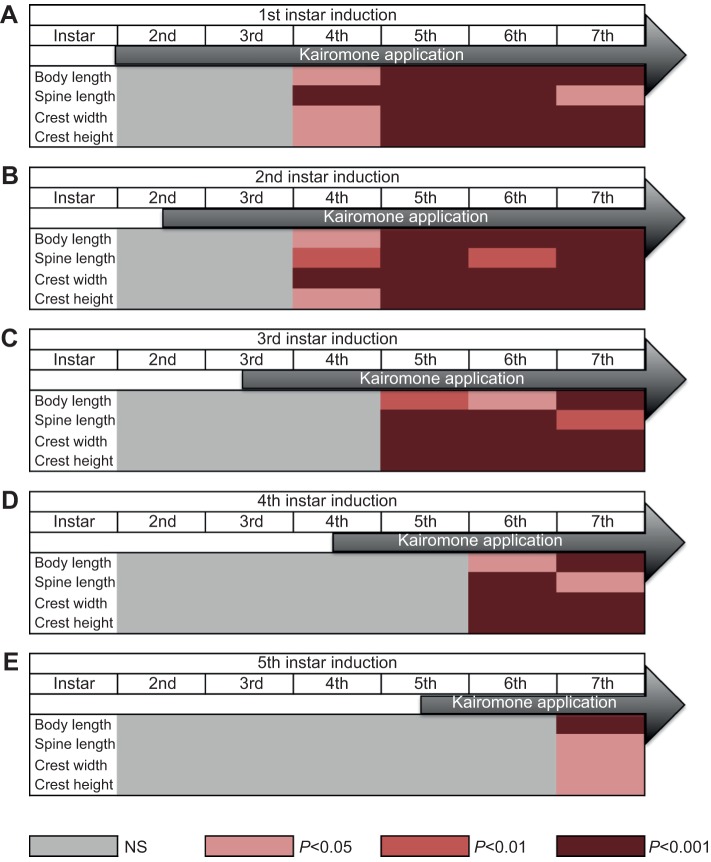
**Kairomone-sensitive stages and time lag of development of inducible defences in *Daphnialongicephala.*** (A–E) Dependence of defence expression on the initiation of kairomone exposure (A: 1st, B: 2nd, C: 3rd, D: 4th and E: 5th instar induction). Levels of significance are shown by shades of red; grey indicates no significant differences (NS) between the kairomone treatment and the control group. Defences are significantly expressed no earlier than the 4th instar irrespective of the initiation of kairomone application, i.e. kairomone exposure in the 1st or in the 2nd instar does not elicit defences before the 4th juvenile instar and therefore kairomone sensitivity starts in the 2nd juvenile instar. From the 2nd juvenile instar, a time lag of one instar can be observed between kairomone exposure and expression of defences. This is valid for all measured kairomone-sensitive stages.

*D. longicephala* that were exposed to *Notonecta* kairomone from the 1st juvenile instar showed significantly larger bodies and tail spines, and developed crests from the 4th juvenile instar compared with control organisms (Table 1, Fig. 1A). All these induced defensive traits developed further in the successive instars if the kairomone was continuously provided (5th, 6th and 7th instar; Table 1, Fig. 1A). No defensive characteristics were observed prior to the 4th instar. Similarly, animals exposed to *Notonecta* kairomone from the 2nd juvenile instar also exhibited the respective defensive traits from the 4th juvenile instar (Table 1, Fig. 1B). Kairomone exposure from the 3rd juvenile instar significantly induced the development of defensive morphological traits in the 5th juvenile instar and onwards (Table 1, Fig. 1C). Accordingly, kairomone exposure in the 4th juvenile instar resulted in the significant increase of the defensive morphological traits in the 6th instar (Table 1, Fig. 1D); and kairomone exposure of animals in the 5th instar induced the development of defensive morphological traits in the 7th instar (Table 1, Fig. 1E).

**Table 1.**
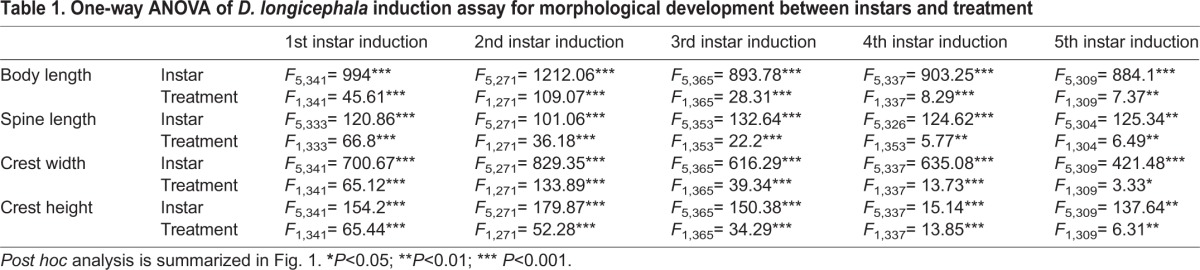
**One-way ANOVA of *D. longicephala* induction assay for morphological development between instars and treatment**

### Additive effects

**Fig. 2.**
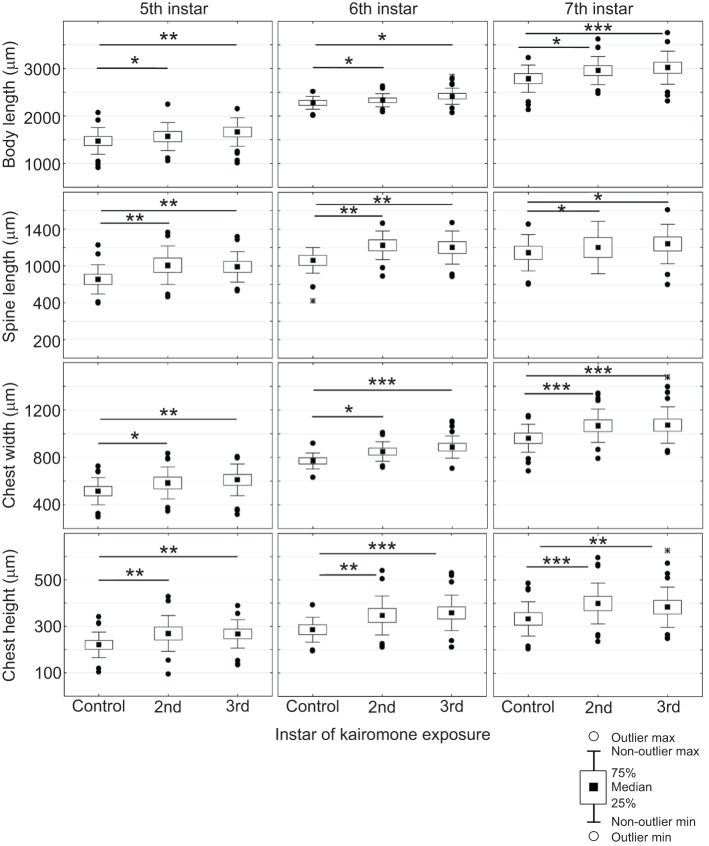
**Effects of short (3rd juvenile instar) and long (2nd juvenile instar) kairomone exposure.** Individual parameters of inducible defences in *D. longicephala* (body length, spine length, crest width and crest height) in the 5th, 6th and 7th instar in control conditions (unexposed to kairomones) and in response to short and long kairomone exposure. Long exposures (beginning in the 2nd juvenile instar) do not induce significantly different inducible defences in comparison to short exposures (from the 3rd instar). Prolonged kairomone application does not result in summation of inducible defence expression. *P≤0.05; **P≤0.01; ***P≤0.001.

In order to determine any additive effect of prolonged kairomone exposure, resulting in larger morphological traits, we compared the expression of traits in the 5th, 6th and 7th instar of *D. longicephala* that had been induced from the 2nd or the 3rd instar. Animals induced from the 2nd instar, which had longer exposure to the kairomone than animals induced from the 3rd instar, did not develop significantly larger morphological traits in the 5th, 6th or 7th instar (Table 2, Fig. 2). Hence, prolonged kairomone exposure does not result in a summation of the cue and therefore does not increase the magnitude of the morphological defence.

**Table 2.**
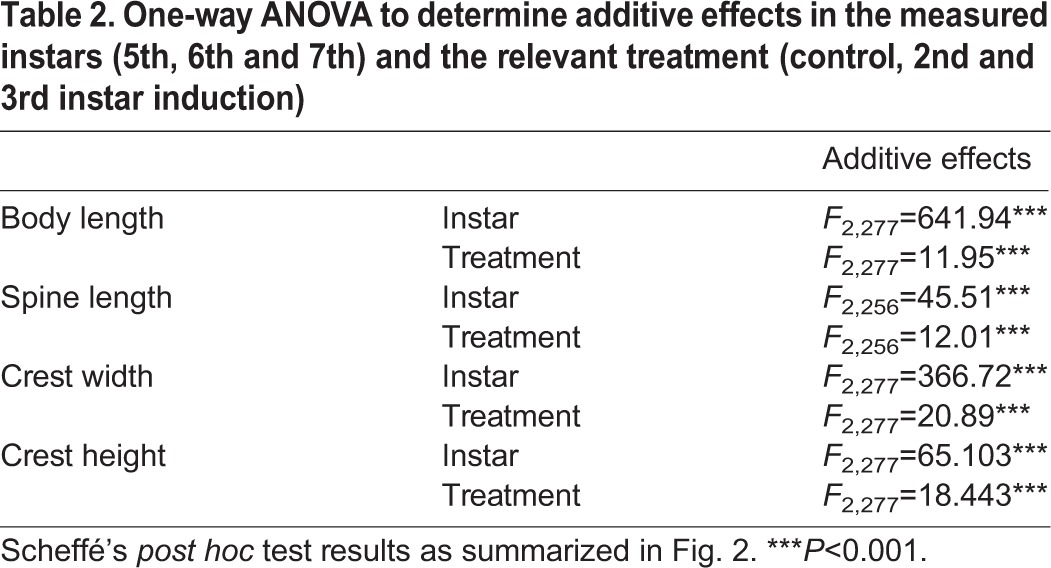
**One-way ANOVA to determine additive effects in the measured instars (5th, 6th and 7th) and the relevant treatment (control, 2nd and 3rd instar induction)**

### Impairment of the first antennae

**Fig. 3.**
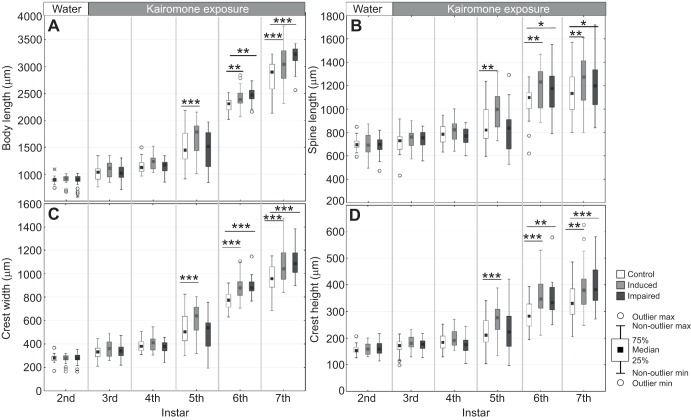
**Location of chemoreceptors for kairomone perception.** Displayed are the individual parameters of inducible defences (A: body length, B: spine length, C: crest width and D: crest height) in *D. longicephala* in the measured instars (2nd to 7th) under control conditions (unexposed - white) and two kairomone treatment groups. One treatment group had intact first antennae (light grey), whereas in the other treatment group first antennae were impaired using underwater adhesive (dark grey). Prior to kairomone exposure, in the 3rd juvenile instar, no significant differences were observed between the three groups for all parameters of inducible defences. In animals with intact first antennae, defences were not significantly developed before the 5th juvenile instar because of the one instar time lag upon induction. Kairomone-treated animals with impaired first antennae showed no significant increase of defences until the 6th juvenile instar. With moulting into the 4th instar, impaired animals shed the underwater adhesive, by which the first antennae were uncovered and reactivated for kairomone detection. This results in significant development of defences in the 6th instar after an induction time lag of one instar. Defensive traits between both treatment groups (intact versus impaired first antennae) are not significantly different thereafter. *P≤0.05; **P≤0.01; ***P≤0.001.

**Fig. 4.**
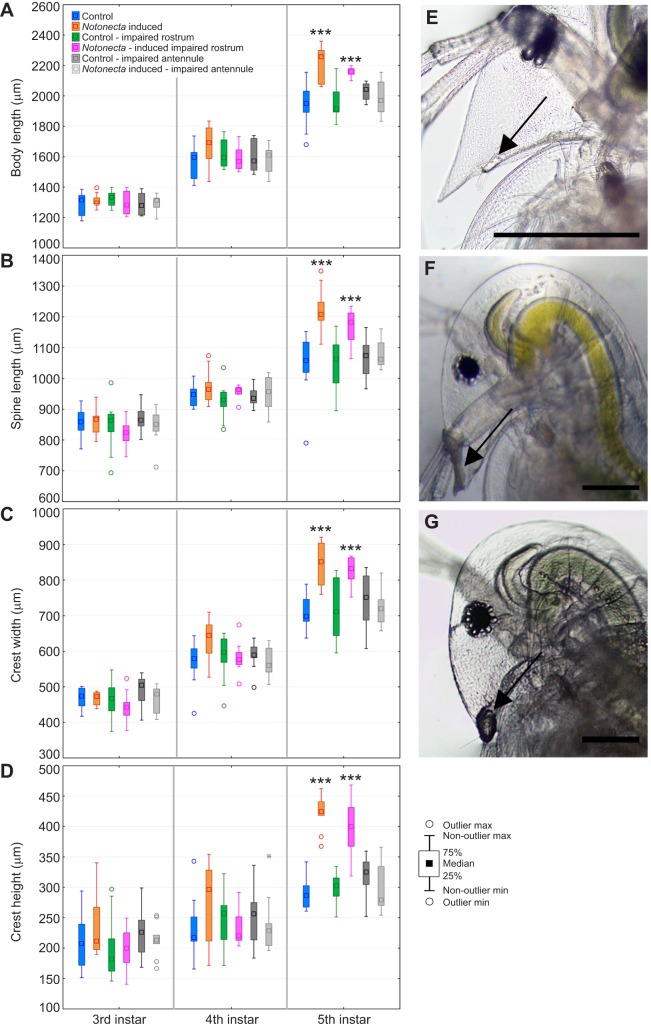
**Control of** **underwater glue side-effects.** (A) Body length, (B) spine length, (C) crest width, (D) crest height measured in control (blue), *Notonecta* induced (orange), control with impaired rostrum (green), induced with impaired rostrum (pink), control with impaired antenna (dark grey) and induced with impaired antenna (light grey). Plotted is median and interquartile range; ***P<0.001. (E) *D. longicephala* with free first antenna (black arrow). (F) In comparison, a *D. longicephala* with an underwater adhesive stuck to the rostral tip (black arrow); with first antenna not covered. (G) First antenna is covered with underwater adhesive (black arrow). Scale bar: 200 µm. See Table 4 for statistical analysis.

As described above, *Notonecta* kairomone exposure starting in the 3rd juvenile instar significantly induced defensive traits in the 5th but not the 4th juvenile instar. These animals kept their defences through the 6th and 7th instar. Animals exposed to the *Notonecta* kairomone with underwater-adhesive-impaired first antennae did not develop defensive traits in the 5th juvenile instar (Fig. 3, Table 3); body length (Fig. 3A), spine length (Fig. 3B), crest width (Fig. 3C) and crest height (Fig. 3D) were not significantly different from the control group. As animals moulted from the 3rd into the 4th instar, the adhesive was shed and the first antennae became uncovered and functional. Congruently with the observations made above, kairomone exposure in the 4th instar induced significant expression of defences in the 6th instar onwards. To test whether the absence of induced defences results from side-effects of the glue, we performed a control experiment in which the glue was applied in the vicinity of the antenna i.e. the tip of the rostrum. Defences in induced animals with glue and without glue were significantly developed (Fig. 4, Table 4). Between these two groups there were no significant differences (Fig. 4, Table 4). Again, animals with a covered antenna did not develop morphological defences in the 5th instar.

**Table 3.**
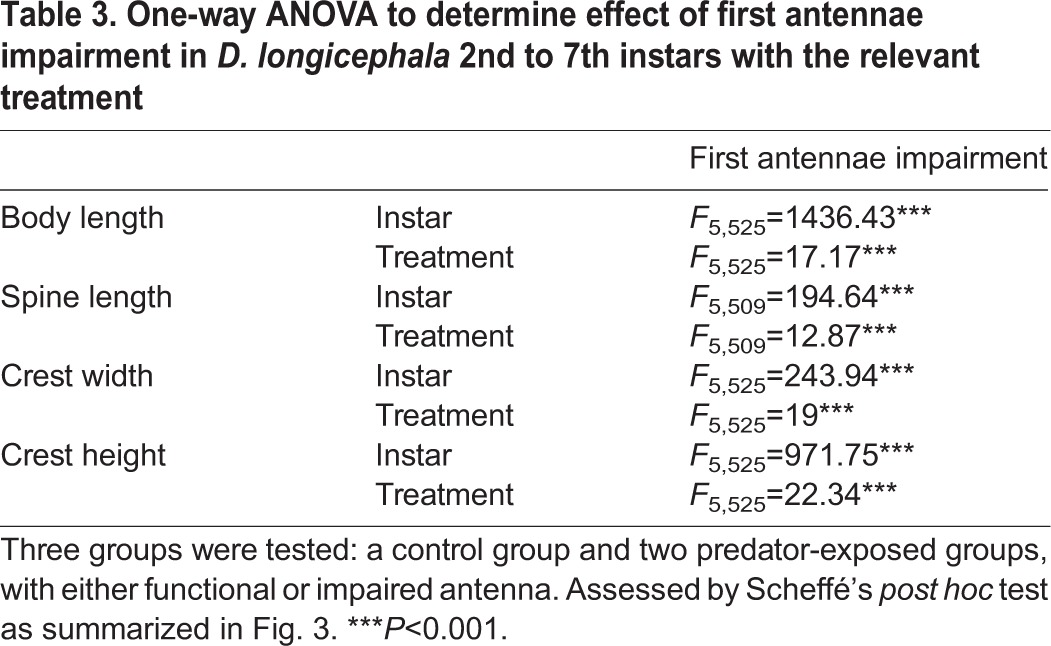
**One-way ANOVA to determine effect of first antennae impairment in *D. longicephala* 2nd to 7th instars with the relevant treatment**

**Table 4.**
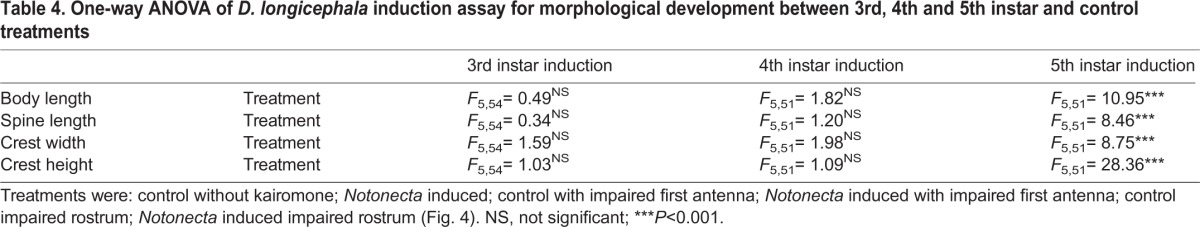
**One-way ANOVA of *D. longicephala* induction assay for morphological development between 3rd, 4th and 5th instar and control treatments**

## DISCUSSION

We have analysed the developmental characteristics of inducible-defence development in *D. longicephala* i.e. (1) kairomone-sensitive stages, (2) developmental time frames, (3) time lags and (4) additive effects resulting from prolonged kairomone exposure. We used this information and developed a method in which we reversibly impaired the first antennae. Our results provide physiological proof that the chemoreceptors for kairomone perception are localized on the first antennae.

### Developmental characteristics of inducible defences in *D. longicephala* and ecological implications

The results obtained in the induction assay show that *D. longicephala* perceive predator cues from the beginning of the 2nd juvenile instar. No effect could be seen from earlier induction. Defensive traits developed with a time lag of one instar, i.e. induction in the 2nd instar led to defence formation in the 4th instar. *N. glauca* is a visual predator that selects larger prey. Juvenile, and therefore smaller, organisms are less likely to be detected and hence do not require defensive traits. Consequently, *D. longicephala* only develop defences in their later life stages i.e. starting from the 4th juvenile instar, when they become beneficial and even essential. In contrast, the period of expression of inducible defences in *D. pulex* is limited to the early embryonic and post-embryonic life stages (Imai et al., 2009; Naraki et al., 2013) as it is vulnerable to the gape-limited predator *Chaoborus* only in early instars (Tollrian, 1995). As such, *D. pulex* are already sensitive to kairomones as embryos (Imai et al., 2009), which also shortens time lags, making defences effective upon birth to assure early protection. In conclusion, different predators require different defensive strategies and the different *Daphnia* species have evolved predation-specific defences appropriate for their encounters in nature.

#### Lack of kairomone sensitivity versus developmental time frames

A sensitive period is a restricted period where an individual is perceptive to specific types of environmental stimuli. Usually this is because of a developmental sensitivity to certain stimuli in that period. The expression of inducible defences can be limited to different developmental time frames, so that they are only present when they are advantageous. This is suspected to be dependent on alternative developmental regimes that can only be turned on or off during defined times in development. Here, we discuss the late onset of expression of defensive traits with respect to kairomone sensitivity versus developmental time frame.

In general, expression of predator-induced defences depends on chemoreception, neuronal signalling and altered developmental pathways. The absence of defence formation in the early juvenile instars (1st to 3rd) could, on the one hand, result from a nervous system not yet able to perceive, integrate or transform the kairomone signal (i.e. dependent on kairomone sensitivity). Although, on the other hand, the neuronally stimulated developmental pathways that result in development of differential morphotypes might not yet be functional (i.e. dependent on a developmental time frame).

In *D. pulex*, neckteeth can be induced as early as the embryonic stages (Laforsch and Tollrian, 2004; Imai et al., 2009; Naraki et al., 2013). This kairomone sensitivity is reported to start with the shedding of the third egg membrane, which liberates the chemosensilla of the first antennae, supposedly allowing chemical perception (Laforsch and Tollrian, 2004). Neckteeth development is then limited to the early juvenile instars.

*D. longicephala* defensive traits, however, cannot be induced before the 2nd instar. If late embryonic or early post-embryonic stages were perceptive to kairomones, defences should be observable before the 4th juvenile instar, as reported for *D. pulex*. However, no significant development was determined in the early (1st to 3rd) instars. In these early instars, the nervous system should already be fully functional and perceptive to chemical cues as the first antennae are not covered, the eyes are fully developed and the nervous system shows normal morphology in the 1st instar (personal observation). Therefore, it is likely that the developmental pathways responsible for development of adaptive morphotypes are not responsive. This means that in *D. longicephala* the expression of defensive traits is limited to certain developmental time frames. The underlying molecular mechanisms, however, still need to be determined.

#### Time lags

In order for inducible defences to be effective, the required time lag preceding the development of defences needs to be as short as possible. Inducible defences in *D. longicephala* require a time lag of one complete instar (e.g. kairomone exposure in the 3rd instar results in defensive traits in the 5th instar, after two moults). Consequently, the underlying time lag is essentially short and defences are expressed maximally 48 h after induction. In juvenile stages these small crustaceans moult daily and the carapace of the next instar is already developing underneath the carapace of the current instar. As such, only the carapace that is developing after induction can undergo morphological change. Developmental programs have already progressed too far to allow a direct kairomone effect on the carapace of the next instar, and so the defences are observed in the subsequent instar.

#### Additive effects

Naraki et al. (2013) showed that the longer *D. pulex* were treated with predator cues, the more neckteeth they developed. This is not the case in this study. Prolonged kairomone stimulation, does not appear to increase the development of defences. Regardless, we cannot exclude additive effects that could be observed when testing lower kairomone concentrations.

### Inactivation and reactivation of the first antennae

In general, chemical cues bind to chemoreceptors. With the help of the information obtained above we aimed to determine the location of the chemoreceptors that bind to *Notonecta* kairomone responsible for the induction of morphological changes. In malacostracan crustaceans, chemoreceptors are centralized on both antennae but not limited to these. Additional chemoreceptors can be found on mouth parts, claws and walking legs (Carr et al., 1987; Breithaupt and Thiel, 2010). In cladocera, the first antennae have been ascribed a chemoreceptive function; however, until now, physiological proof was missing. The inactivation of the first antennae in the 3rd juvenile instar using underwater adhesive hindered the development of defensive traits in the 5th instar. This suggests that animals were not able to perceive the predator cues. In conclusion, the receptors for the detection of the *Notonecta* kairomone must be located on the first antennae. With the shedding of the carapace when moulting from the 3rd into the 4th instar, animals also shed the adhesive, which liberated the first antennae. Organisms were then perceptive to kairomones in the 4th instar, as we observed the development of defences in the 6th instar. We can rule out that the observed effect results from side effects caused by the adhesive. The control application of the adhesive to the rostrum did not impair development of morphological defences in the 5th instar (Table 4, Fig. 4). This shows that the first antennae carry the receptors for kairomone perception. The absence of defences in the 5th instar cannot result from the treatment itself, as we were able to reactivate the defence formation through the liberation of the first antennae in the 4th instar, resulting in significant development of traits in the 6th instar.

### Conclusion

As an environmental keystone species, *Daphnia* has been the focus of ecological research for decades. In particular, its sensitivity and responsiveness to a vast diversity of environmental cues has attracted interest from researchers from different disciplines in biological research. In the era of next-generation sequencing and growing interest in ‘environmental omics’, *Daphnia* is now popularly used to investigate how the environment affects genomes, transcriptomes, epigenomes, metabolomes etc. The description of how *Daphnia* perceive their environment is fundamental for any kind of ‘environmental omic’ approach. Our results give a deeper insight into this environmental perception and development of adaptive features, for which it has been ecologically investigated throughout the past decades.

## MATERIALS AND METHODS

All experiments were performed using a *Daphnia longicephala* (Hebert 1977) clone, which originated from Lara Pond, Australia.

### Animal cultures

#### *Daphnia longicephala* culture

Age-synchronized daphniids were cultured in 1 litre beakers (WECK^®^, Wehr, Germany) filled with charcoal-filtered tap water under standardized conditions (20±0.1°C; 12 h:12 h light:dark cycle) in an incubator (KBF 750, Binder; Tuttlingen, Germany). Populations were restricted to 30–40 individuals per beaker to prevent the production of males and resting eggs. The animals' exuviae and algal remains were removed from the beaker and charcoal-filtered tap water was exchanged regularly. Daphniids were fed *ad libitum* with the algae *Scendesmus obliquus*.

#### *Notonecta* culture

*Notonecta* from the ponds of the Ruhr University Botanical Gardens were used for kairomone production. They were maintained in charcoal-filtered tap water under standardized conditions (20±1°C with a 16 h:8 h light:dark cycle). Notonectids were exclusively cultivated for kairomone production and regularly fed with daphniids *ad libitum*.

#### Preparation of *Notonecta* kairomone

For production of the kairomone, one adult *Notonecta* and 10 adult *D. longicephala* were transferred into 200 ml charcoal-filtered tap water for incubation under standardized conditions (20°C±1°C; 12 h:12 h light:dark cycle). After 24 h, when the *Notonecta* had consumed all daphniids, *Notonecta* and remnants of dead *Daphnia* were removed. In order to prevent bacterial degradation, the medium was filtered (0.45 μm GF/C Whatman, Sigma-Aldrich, Germany) and 10 mg l^−1^ of the antibiotic ampicillin (Ampicillin Sodiumsalt, K029.1, Carl ROTH, Karlsruhe, Germany) was added. Ampicillin does not affect *Daphnia* defences and/or development. The kairomone was stored at −20°C until further usage. This stock concentration of 5 *Notonecta* per litre was diluted (1:5) to 1 *Notonecta* per litre using charcoal-filtered tap water for use in the experiments.

### Induction of morphological defences

**Fig. 5.**
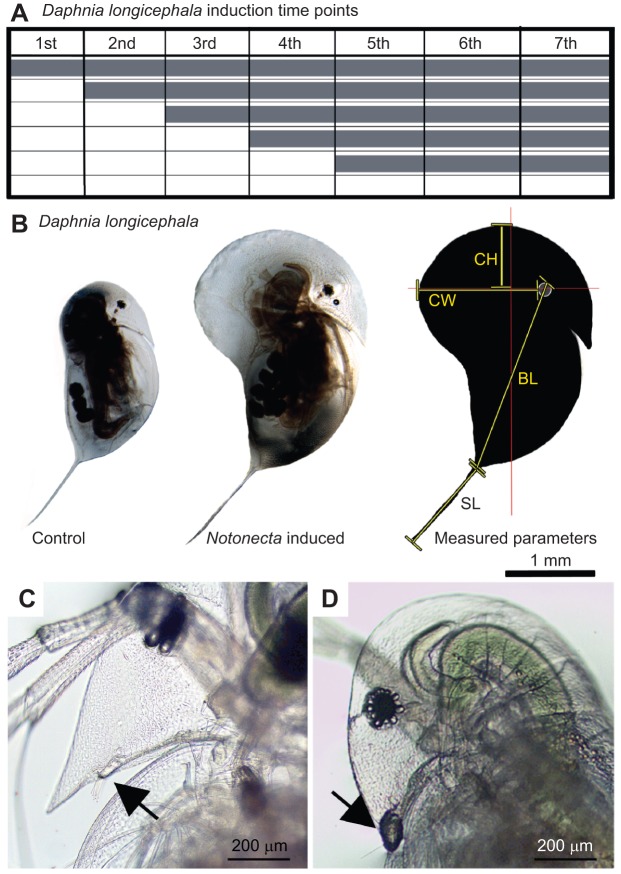
**Experimental procedures.** (A) *D. longicephala* induction scheme. Animals were exposed to *Notonecta* kairomone starting in the 1st, 2nd, 3rd, 4th and 5th juvenile instars respectively (grey bars). Controls were performed in the absence of the kairomone. Photographic documentation started in the 2nd juvenile instar and was continued for all successive instars until animals deposited their 2nd clutch (7th instar). (B) Measurement of defences in *D. longicephala.* Control (left) and predator-exposed (middle) *D. longicephala*. Right panel shows measured parameters that quantify morphological defences (yellow lines) with respect to the body axis (red lines). BL, body length; SL, spine length; CH, crest height; CW, crest width. (C) First antennae of a female *D. longcicephala* (black arrow). (D) First antennae of a female *D. longicephala* covered with underwater glue.

Animals in the experiments were reared in a climate incubator with a standard temperature of 20°C (±0.1°C) and a 12 h:12 h light:dark cycle. Newborn daphniids (1st juvenile instar) from *D. longicephala* maintained in standard conditions, were placed individually in 50 ml glass vials containing either 20 ml charcoal-filtered tap water (control) or 20 ml kairomone (1 *Notonecta* per litre). As the diluted kairomone stock (see above) included 2 mg ampicillin l^−1^, this was also added to the control vessels in order to ensure similar experimental conditions. The algae *S. obliquus* was added as a food source at a concentration of 1.5 g l^−1^. Induction of defensive traits was assessed with a full-factorial experimental set-up consisting of six treatments, differentiated by the onset of kairomone exposure. Within each synchronized experiment, each of the 6 treatments was replicated in parallel 5 times. Subsequently, this full-factorial experimental set-up (consisting of the treatments and synchronized replicates) was replicated successively 8 times. No significant differences were observed between these successive replicates, which allowed us to pool the data for each treatment. Therefore, the overall number of replication for each treatment is *N*=40. *D. longicephala* neonates were continuously reared for seven instars in charcoal-filtered tap water (with 2 mg Amp l^−1^). The five exposure groups were transferred from this charcoal-filtered tap water to the kairomone stock in the 1st, 2nd, 3rd, 4th and 5th juvenile instar, respectively, and maintained in kairomone stock thereafter (Fig. 5A). All media were exchanged regularly every 48 h to maintain stable culture conditions. Daphniids were photographed using a stereomicroscope (Olympus SZX16) equipped with a digital camera (Color View III digital imaging system) and imaging software (Cell^D; Soft Imaging Solutions, SIS Olympus, Münster, Germany) in the 2nd, 3rd, 4th and 5th juvenile instar, and following moulting in the 6th and 7th instar (in which they deposited their first and second clutch). Induced defences – body length, tail spine length, crest width and crest height – were measured from the photos to compare control with kairomone-induced organisms (Fig. 5B). Body length was determined from the upper margin of the eye to the junction of carapace and tail spine. Tail spine length extended from the carapace junction to the tip of the spine. Crest width was measured from the dorsal eye margin to the dorsal point of dorsal crest extension determined and crest height from the upper eye to the distal height of the crest, rectangularly to the crest width and parallel to the body axis (Fig. 5B). To enhance statistical power, this experimental set-up was repeated eight times using identical procedures.

### Impairment of the first antennae

First antennae were impaired by carefully placing *D. longicephala* on object slides. Residual water was removed and daphniids were further dried using filter paper. Under a dissection microscope we placed a drop of underwater adhesive (transparent underwater adhesive by Hobby Aquaristik, Germany) on each of the first antenna. By pulling Pasteur pipettes (VWR, Germany) over a hot flame, we produced fine glass capillaries with which we carefully covered the paired first antennae with the adhesive (Fig. 5D). Careful attention was taken not to harm the daphniids during adhesive application. The whole process was performed quickly (<1 min), and daphniids were placed in 50 ml glass vials holding 40 ml diluted *Notonecta* kairomone stock (described above). In order to test whether the first antennae are responsible for kairomone detection, animals with impaired antennae (Fig. 5D) were compared with a control group, reared in charcoal-filtered tap water and a group reared in *Notonecta* kairomone with functional antennae (Fig. 5C). All three treatments were photographed in order to document body length, spine length, crest height and crest width in the 5th instar. All animals, including those with the underwater adhesive, behaved normally and were able to moult into subsequent instars in synchronization with the animals of kairomone-exposed and control groups. During the next moult, the adhesive was shed with the exuviae. No residual adhesive could be observed and the first antennae looked normal and healthy. In order to control for side-effects that may be caused by the underwater adhesive, we performed an assay in which we compared control and induced animals with and without impaired antenna to control and induced animals that had a similar amount of glue applied in close vicinity of the first antennae, i.e. the rostral tip. Each treatment was replicated 10 times. We determined body length, spine length, crest width and crest height in the 3rd, 4th and 5th juvenile instar as described above (Fig. 4, Table 4). No such side-effects were noticed.

### Data analysis

Statistical analyses were performed using the software Statistica 10 (Statsoft Incorporation, USA). Data followed a normal distribution with equal variances and therefore were analysed using a one-way analysis of variance (ANOVA). Differences between individual groups were determined using a Scheffé *post hoc* analysis.
